# Analysis of Histopathological Lesions and Their Correlation With Upper Gastrointestinal Endoscopic Findings: A Retrospective Study at a District Hospital in Ghana

**DOI:** 10.1002/hsr2.72741

**Published:** 2026-06-30

**Authors:** Prosper Adjei, Samuel Kyeremeh Adjei

**Affiliations:** ^1^ Department of Internal Medicine Methodist Hospital Wenchi Ghana

**Keywords:** Biopsy, Endoscopy, Ghana, Histopathology, Upper Gastrointestinal

## Abstract

**Background and Aims:**

Although upper gastrointestinal (GI) endoscopy is a crucial diagnostic tool for evaluating lesions of the upper GI tract, its findings are not always definitive, necessitating histopathological confirmation. This retrospective study aimed to assess the spectrum of histopathological lesions and their correlation with endoscopic impressions at Methodist Hospital, Wenchi, Ghana.

**Methods:**

A retrospective observational study was conducted on patients who underwent upper GI endoscopy with biopsy between August 2024 and September 2025. Out of 513 patients who underwent upper GI endoscopy, 176 were biopsied, but 46 were excluded due to missing histopathology reports, leaving 130 patients for analysis. Endoscopic impressions were compared with histopathological diagnoses across different GI tract sites (esophagus, stomach, and duodenum). Diagnostic accuracy was calculated with sensitivity, specificity, and 95% confidence intervals (CIs). Agreement was assessed using Cohen's Kappa and prevalence‐adjusted bias‐adjusted Kappa (PABAK).

**Results:**

Of the 130 patients, 97.7% of lesions were benign (predominantly chronic gastritis), and 2.3% were malignant. Endoscopy demonstrated a sensitivity of 100% (95% CI: 29.2–100) and specificity of 99.2% (95% CI: 95.8–100) for malignant lesions. Cohen's Kappa was 0.796, indicating substantial agreement, while PABAK was 0.966, reflecting near‐perfect concordance after adjusting for prevalence.

**Conclusion:**

Upper GI endoscopy showed high diagnostic accuracy and substantial concordance with histopathology for benign lesions such as gastritis. These findings suggest that clinicians in resource‐deprived areas where histopathology is not readily available or may be associated with long turnaround times can safely commence targeted treatments for benign upper GI conditions based primarily on endoscopic findings.

**Abbreviations**
df: Degree of Freedom
ESCC: Esophageal Squamous Cell Carcinoma
GI: Gastrointestinal
NPV: Negative Predictive Value
PABAK: Prevalence‐Adjusted Bias‐Adjusted Kappa
PPV: Positive Predictive Value
SD: Standard Deviation
SPSS: Statistical Package for Social Sciences
X^2^
: Chi‐square

## 1 Introduction

The gastrointestinal (GI) tract is a common site for a range of lesions, which include congenital, inflammatory, and neoplastic conditions [1]. Disorders of the GI tract are among the most frequently encountered issues in clinical practice and contribute significantly to morbidity and mortality [2]. Upper GI endoscopy has emerged as a cornerstone in the evaluation of patients presenting with upper GI symptoms [3]. It is a widely recognized procedure for the diagnosis and management of various gastric and duodenal lesions [4]. Nonetheless, endoscopic findings are often not pathognomonic because different conditions can have similar appearances, necessitating histopathological confirmation.

Upper GI endoscopy, along with biopsy, is crucial for diagnosing and managing a range of GI tract diseases [5]. Endoscopic biopsy allows for direct visualization and targeted sampling of suspicious areas, enabling the early detection and accurate diagnosis of pathological conditions [6]. Additionally, endoscopic biopsies help to assess the extent of GI tract diseases and response to treatment, monitor disease progression, and enable early detection of complications [7].

Histopathological analysis of endoscopic biopsies provides essential information regarding the characteristics and severity of GI disorders, thereby informing clinical decision‐making and therapeutic approaches [8].

Since the introduction of upper GI endoscopy to Ghana over four decades ago, few studies correlating endoscopic findings with histopathological diagnoses have been conducted at some tertiary centers in the country. Due to the unavailability of essential diagnostic services in most district hospitals, where the majority of patients first seek medical care, there is limited evidence regarding how endoscopic impressions align with histopathological findings in these settings. The paucity of data carries significant clinical implications, which include potential misdiagnosis, delayed initiation of appropriate therapy, delayed oncology referrals, and unwarranted interventions. In our earlier publication in Health Science Reports [3], we described the spectrum of endoscopic findings among 396 patients at Methodist Hospital, Wenchi, Ghana, but that study did not incorporate histopathological correlation. The present study builds directly upon that work by analyzing a new cohort of 130 patients (August 2024–September 2025), distinct from the previously reported group, and explicitly linking endoscopic impressions with histopathological diagnoses. This directly addresses a critical diagnostic gap, ensuring quality healthcare delivery and improving patient outcomes in resource‐constrained district hospitals across Ghana.

## 2 Materials and Methods

### 2.1 Study Design and Setting

We conducted a retrospective review to evaluate upper GI lesions and examine how endoscopic impressions aligned with histopathological findings at Methodist Hospital, Wenchi, Ghana, over a period extending from August 2024 to September 2025. The hospital is a primary level facility with six different specialties and also serves as the principal referral facility for the Wenchi Municipality and its environs.

### 2.2 Patient Selection (Inclusion and Exclusion Criteria)

**Figure 1:**
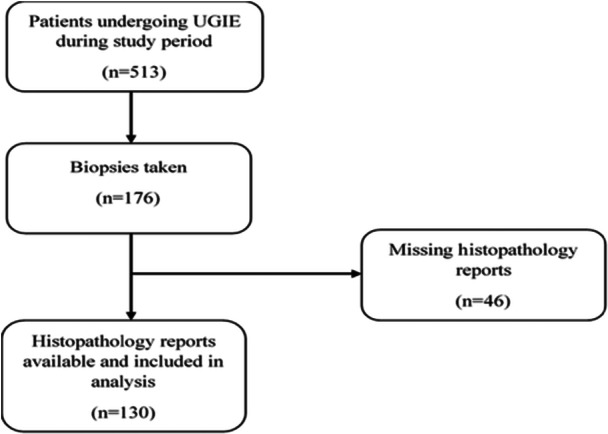
Flow chart of patient inclusion and exclusion. Abbreviation: UGIE, Upper gastrointestinal endoscopy.

The study population consisted of patients who underwent upper GI endoscopy and biopsy at the endoscopy unit, with available histopathological reports during the study period. Participants with incomplete clinical data or missing histopathological reports were excluded from the study (Figure 1).

### 2.3 Endoscopic Procedure

Endoscopic procedures were performed by a physician specialist with training and certification in endoscopy following established protocols using Sonoscape HD 500 Endoscope‐EG‐500L (Sonoscape Medical Corporation, Resolution‐1080 pixel, Full HD:60FPS, HDL‐500X Xenon light source, VIST Chromoendoscopy). Prior to the procedure, all patients fasted for a minimum of 6 h and underwent a thorough pre‑procedural assessment. For local anesthesia, the pharynx was sprayed with 10% lignocaine to improve comfort. Sedation was achieved using 2.5 mg of intravenous midazolam, while intravenous hyoscine butylbromide was administered to facilitate smooth muscle relaxation and reduce GI spasms. Biopsies were obtained from identified lesions in the esophagus, stomach, and duodenum, using standard (oval cup) biopsy forceps. For suspected gastritis, biopsies were obtained in accordance with the updated Sydney protocol, with samples taken from the antrum (2 samples), corpus (2 samples), and incisura angularis (1 sample). For ulcers and suspected malignancies, six to eight biopsy specimens were taken to ensure high diagnostic accuracy. Samples were obtained from both the edges and the base of the ulcers. Biopsy samples were kept in 10% formaldehyde solution and transported to an external laboratory for histopathological examination.

### 2.4 Histopathological Examination

Multiple pathologists examined the biopsy specimens histopathologically in accordance with standardized protocols. Each sample was grossly examined, processed through dehydration, clearing, and paraffin embedding, and sectioned at 3–5 µm thickness using a microtome. Slides were stained routinely with hematoxylin and eosin (H&E), with additional special stains applied when indicated. Microscopic evaluation was performed by pathologists to assess tissue architecture, cellular morphology, and the presence of inflammatory, premalignant, or malignant changes. Final diagnoses were documented in standardized histopathology reports.

### 2.5 Statistical Analysis

Data analysis was carried out using SPSS version 25. Associations between endoscopic and histological findings were tested with Chi‐square, with *p*‐values < 0.05 considered significant. Cohen's Kappa analysis was used to assess the agreement between endoscopic and histopathological findings. Prevalence‐adjusted bias‐adjusted Kappa (PABAK) was additionally calculated to provide a more robust measure of concordance.

### 2.6 Ethical Approval

Ethical approval for the study was obtained from the Research Ethics Committee of Methodist Hospital, Wenchi, Ghana (Ethics approval reference: MHW/RP/103/01). Informed consent was waived due to the retrospective nature of the study and patients' data were anonymized to protect confidentiality.

## 3 Results

### 3.1 Socio‐Demographic and Clinical Characteristics of Participants

A total of 513 patients underwent upper GI endoscopy during the study period, from which 176 biopsies were taken for histopathological examination. Of these, 130 histopathology reports were available for analysis.

The mean age of participants was 47.9 ± 17.5 years with an age range of 13 to 96 years. The majority of participants (29.2%, *n* = 38) were aged ≥ 60 years, followed by 40–49 years (20.0%, *n* = 26) and 30–39 years (19.2%, *n* = 25). Participants younger than 20 years represented only 4.6% (*n* = 6) of the study population. Females constituted a slightly higher proportion (53.8%, *n* = 70) compared to males (46.2%, *n* = 60). The predominant indication for endoscopy was epigastric pain (81.5%, *n* = 106), followed by upper GI bleeding (6.2%, *n* = 8) and retrosternal chest pain (4.6%, *n* = 6). Other less frequent indications included epigastric mass, heartburn, sensation of a foreign body in the throat, liver cirrhosis, and dysphagia. The stomach was the most common biopsy site (93.1%, *n* = 121), whereas the duodenum and esophagus accounted for 6.2% (*n* = 8) and 0.8% (*n* = 1), respectively. *Helicobacter pylori* stool antigen test results were available for 118 patients, with 98 positives, yielding an overall prevalence of 83.1% (Table 1).

**Table 1:** Socio‐demographic and clinical characteristics of participants.

Variable	Frequency (*N*)	Percentage (%)
Age (years)		
Mean(± SD)	47.9 ± 17.5	
Range	13–96	
Age group		
< 20	6	4.6
20–29	15	11.5
30–39	25	19.2
40–49	26	20.0
50–59	20	15.4
≥ 60	38	29.2
Total	**130**	**100**
Gender		
Male	60	46.2
Female	70	53.8
Total	**130**	**100**
Indication for Endoscopy		
Epigastric pain	106	81.5
Epigastric mass	1	0.8
Retrosternal chest pain	6	4.6
Upper GI bleeding	8	6.2
Heartburn	2	1.5
Sensation of a foreign body in the throat	4	3.1
Liver Cirrhosis	2	1.5
Dysphagia	1	0.8
Total	**130**	**100**
Biopsy site		
Esophagus	1	0.8
Stomach	120	92.3
Duodenum	9	6.9
Total	**130**	**100**
Endoscopic Diagnosis		
Esophageal cancer	1	0.8
Gastric cancer	3	2.3
Gastric ulcer	11	8.5
Gastritis	97	74.6
Duodenal ulcer	7	5.4
Duodenitis	2	1.5
Normal	9	6.9
Total	**130**	**100**
Histology Diagnosis		
Adenocarcinoma	1	0.8
Squamous cell carcinoma	2	1.5
Benign ulcer	9	6.9
Chronic gastritis	75	57.7
Chronic gastritis with intestinal metaplasia	13	10.0
Gastric erosion	1	0.8
Chronic duodenitis	3	2.3
Normal gastric biopsy	25	19.2
Normal duodenal biopsy	1	0.8
Total	**130**	**100**
Classification of Lesions		
Benign	127	97.7
Malignant	3	2.3
Total	**130**	**100**

Abbreviations: GI, gastrointestinal; SD, standard deviation.

Table 2 shows the predominant endoscopic features of the various GI lesions detected in the participants.

**Table 2:** Predominant endoscopic features of gastrointestinal lesions detected in the participants.

Lesion	Endoscopic features
Esophageal cancer	Large, fungating mass completely occluding the lumen; bleeds easily on contact
Gastric cancer	Irregular, ulcerated, or fungating lesion with friable mucosa
Gastric ulcer	Well‐demarcated excavation; base covered by white exudate or fibrin
Mild gastritis	Patchy or minimal localized mucosal erythema with or without mucosal edema (mild)
Moderate gastritis	Widespread patchy or diffuse mucosal erythema; moderate mucosal edema
Severe gastritis	“Beefy” red discoloration involving an extensive area or the entire gastric mucosa; pronounced mucosal edema; friable mucosa
Duodenal ulcer	Well‐demarcated, oval or round lesion; base covered by white exudate
Duodenitis	Mucosal erythema; friable mucosa

### 3.2 Concordance Between Endoscopic and Histopathological Diagnoses

#### 3.2.1 Esophagus

The endoscopic diagnosis of esophageal cancer demonstrated a 100% concordance rate (*n* = 1) with histopathological findings, which confirmed squamous cell carcinoma, underscoring the diagnostic reliability of endoscopy in this case (Table 3).

**Table 3:** Concordance between endoscopic and histological diagnoses of esophageal biopsies.

Endoscopic findings	Histopathological findings	Total (*N*) (%)
	Squamous cell carcinoma (*N*) (%)	
Esophageal cancer	1 (100)	1 (100)
Total	1 (100)	1 (100)

#### 3.2.2 Stomach

Among the three cases endoscopically suspected to be gastric cancer, histological examination confirmed one as adenocarcinoma and another as squamous cell carcinoma, while the third was identified as a benign lesion, indicating a good concordance. Gastric ulcer showed a concordance rate of 54.5% (*n* = 6), whereas gastritis demonstrated a high concordance rate of 82.5% (*n* = 80) (Table 4).

**Table 4:** Concordance between endoscopic and histopathological diagnoses of gastric biopsies.

Endoscopic findings	Histopathological findings							Total (*N*) (%)
	Benign findings					Malignant findings		
	Benign ulcer (*N*) (%)	Gastric erosion (*N*) (%)	Chronic gastritis (*N*) (%)	Chronic gastritis with intestinal metaplasia (*N*) (%)	Normal gastric biopsy (*N*) (%)	Adenocarcinoma (*N*) (%)	Squamous cell carcinoma (*N*) (%)	
Normal	0 (0.0)	0 (0.0)	1 (11.1)	0 (0.0)	8 (88.9)	0 (0.0)	0 (0.0)	9 (7.5)
Gastric cancer	0 (0.0)	0 (0.0)	1 (33.3)	0 (0.0)	0 (0.0)	1 (33.3)	1 (33.3)	3 (2.5)
Gastric ulcer	6 (54.5)	1 (9.1)	4 (36.4)	0 (0.0)	0 (0.0)	0 (0.0)	0 (0.0)	11 (9.2)
Gastritis	0 (0.0)	0 (0.0)	67 (69.1)	13 (13.4)	17 (17.5)	0 (0.0)	0 (0.0)	97 (80.8)
Total	6 (5.0)	1 (0.8)	73 (60.8)	13 (10.8)	25 (20.8)	1 (0.8)	1 (0.8)	120 (100)

#### 3.2.3 Duodenum

The endoscopic diagnosis of duodenal ulcer had a 42.9% (*n* = 3) concordance rate with histopathological findings. Duodenitis exhibited moderate agreement at 50.0% (*n* = 1) (Table 5).

**Table 5:** Concordance between endoscopic and histological diagnoses of duodenal biopsies.

Endoscopic findings	Histopathological findings				Total (*N*) (%)
	Benign ulcer (*N*) (%)	Chronic duodenitis (*N*) (%)	Chronic gastritis (*N*) (%)	Normal duodenal biopsy (*N*) (%)	
Duodenal ulcer	3 (42.9)	2 (28.6)	1 (14.3)	1 (14.3)	7 (77.8)
Duodenitis	0 (0.0)	1 (50.0)	1 (50.0)	0 (0.0)	2 (22.2)
Total	3 (33.3)	3 (33.3)	2 (22.2)	1 (11.1)	9 (100)

### 3.3 Diagnostic Accuracy of Endoscopy

Endoscopic diagnosis demonstrated high accuracy for both malignant and benign lesions. Sensitivity for detecting malignancy was 100% (95% CI: 29.2–100), with specificity at 99.2% (95% CI: 95.8–100). The positive predictive value (PPV) for malignancy was 75%, while the negative predictive value (NPV) was 100%. For benign lesions, sensitivity was 99.2% (95% CI: 95.8–100) and specificity was 100% (95% CI: 29.2–100). The PPV and NPV for benign lesions were 100% and 75%, respectively. Overall diagnostic accuracy for both categories was 99.2% (Table 6). When diagnostic accuracy was stratified by age, gender, and biopsy site, several important patterns emerged. Malignancy was confined to patients aged ≥ 60 years, where endoscopy achieved perfect sensitivity (100%) but slightly lower specificity (97.1%) due to one false‐positive impression, yielding a PPV of 75%. In contrast, younger age groups (< 60 years) had exclusively benign lesions, resulting in perfect concordance between endoscopic and histological diagnoses. Gender‐based stratification revealed flawless diagnostic accuracy among females (100%), while males demonstrated slightly reduced specificity (98.3%) owing to a single false‐positive malignancy. Site‐specific analysis showed perfect accuracy for esophageal lesions and very high accuracy for gastric biopsies (99.2%), whereas duodenal lesions exhibited lower concordance (88.9%) (Table 7).

**Table 6:** Diagnostic accuracy of endoscopy, taking histopathology as a gold standard diagnostic tool.

Statistic	Malignancy	Benign
Sensitivity	100% (95% CI: 29.2–100)	99.2% (95% CI: 95.8–100)
Specificity	99.2% (95% CI: 95.8–100)	100% (95% CI: 29.2–100)
Positive Predictive Value	75%	100%
Negative Predictive Value	100%	75%
Accuracy	99.2%	99.2%

Abbreviation: CI, Confidence interval.

**Table 7:** Stratified subgroup analysis of diagnostic accuracy of endoscopy taking histopathology as gold standard.

Subgroup	Sensitivity (%)	Specificity (%)	PPV (%)	NPV (%)	Accuracy (%)
Age group (years)					
< 20 (*n* = 6)	100	100	100	100	100
20 – 39 (*n* = 40)	98.0	100	100	97.5	98.5
40 – 59 (*n* = 46)	98.0	100	100	97.8	98.9
≥ 60 (n = 38)	100	97.1	75.0	100	97.4
Gender					
Male (*n* = 60)	100	98.3	75.0	100	98.3
Female (*n* = 70)	100	100	100	100	100
Biopsy site					
Esophagus (*n* = 1)	100	100	100	100	100
Stomach (*n* = 120)	99.2	99.2	75.0	100	99.2
Duodenum (*n* = 9)	85.7	88.9	75.0	88.9	88.9

Abbreviations: NPV, Negative Predictive Value; PPV, Positive Predictive Value.

### 3.4 Association Between Endoscopic Findings and Histopathological Diagnoses of Gastric Biopsies

An analysis of the association between endoscopic impressions and histopathological findings of gastric biopsies revealed a strong diagnostic alignment across the various categories. Among the nine cases with normal endoscopic findings, histology confirmed normal gastric mucosa in eight cases (88.9%), while one case (11.1%) showed benign pathology. In the group of 108 cases endoscopically diagnosed as benign conditions, histopathological evaluation confirmed benign lesions in all cases (100%), demonstrating excellent concordance and suggesting strong diagnostic accuracy of endoscopy for non‐malignant gastric conditions. For the three cases suspected to be malignant based on endoscopic appearance, histology confirmed malignancy in two cases (66.7%), while one case (33.3%) was found to be benign. Statistical analysis using Chi‐square test revealed a significant association between endoscopic and histopathological diagnoses (*X* ^2^ = 1, *p* < 0.001) (Table 8).

**Table 8:** Association between endoscopic and histopathological findings of gastric biopsies.

Endoscopic findings	Histopathological findings				*X* ^2^ (df)	*P*‐value
	Normal (*N*) (%)	Benign (*N*) (%)	Malignant (*N*) (%)	Total		
Normal	8 (88.9)	1 (11.1)	0 (0.0)	9 (7.5)		
Benign	0 (0.0)	108 (100)	0 (0.0)	108 (90.0)	1	0.001
Malignant	0 (0.0)	1 (33.3)	2 (66.7)	3 (2.5)		
Total	8 (6.7)	110 (91.7)	2 (1.7)	120 (100)		

Abbreviation: df, Degree of freedom.

Cohen's Kappa analysis indicated a substantial level of agreement between endoscopic and histological diagnoses, with a Kappa coefficient of 0.796 (*p* = 0.001). Additionally, PABAK was 0.966, reflecting near‐perfect agreement after accounting for the extreme prevalence of benign lesions (Table 9).

**Table 9:** Cohen's Kappa and prevalence‐adjusted bias‐adjusted Kappa analysis.

Endoscopic findings	Histopathological findings				Kappa coefficient	*P*‐value	PABAK
	Normal (*N*) (%)	Benign (*N*) (%)	Malignant (*N*) (%)	Total			
Normal	8 (88.9)	1 (11.1)	0 (0.0)	9 (7.5)			
Benign	0 (0.0)	108 (100)	0 (0.0)	108 (90.0)	0.796	0.001	0.966
Malignant	0 (0.0)	1 (33.3)	2 (66.7)	3 (2.5)			
Total	8 (6.7)	110 (91.7)	2 (1.7)	120 (100)			

Abbreviation: PABAK, Prevalence‐Adjusted Bias‐Adjusted Kappa.

## 4 Discussion

While endoscopy serves as the main diagnostic modality for upper GI pathologies, histopathological confirmation may be necessary to ensure greater diagnostic precision in certain cases. This study sought to evaluate the spectrum of histopathological lesions and their correlation with upper GI endoscopic findings at a resource‐deprived district hospital in Ghana. The majority of participants who underwent upper GI endoscopy with biopsy were aged 60 years or older, and females constituted a slightly higher proportion. Overall, benign lesions predominated (97.7%), with chronic gastritis being the most frequent diagnosis, while malignancy was rare (2.3%). Endoscopy demonstrated high sensitivity and specificity, as well as substantial concordance (Cohen's Kappa 0.796; PABAK 0.966), with histopathological diagnoses.

In the present study, most of the participants were aged 60 years or older, consistent with findings from a previous study that reported a similar trend [9]. Elderly patients in Ghana frequently take medications for multiple chronic medical conditions, which potentially exposes their gastric mucosa to the damaging effects of polypharmacy. Again, older patients presenting with upper GI symptoms are more likely to have age‐related “alarm features” compared to younger individuals, thus making clinicians more proactive in scheduling them for endoscopic assessment. Regarding gender distribution, the majority of biopsies were obtained from female participants (53.8%, *n* = 70). This finding is consistent with the report by Choomsri et al., who also observed a predominance of females [10]. Conversely, several other studies reported male preponderance [11, 12, 13, 14, 15]. The observed female preponderance in this study may be partly explained by gender differences in health‐seeking behavior. Women are generally more attuned to bodily changes and more likely to report GI symptoms to healthcare providers. This proactive approach often leads to earlier and more frequent referrals for endoscopic evaluation. Additionally, women tend to demonstrate greater compliance with follow‐up appointments and screening programs, further increasing their likelihood of undergoing biopsy procedures and contributing to their over‐representation in research reports.

The current study revealed a high prevalence of benign lesions in the upper GI tract (97.7%), which agrees with earlier studies highlighting the predominance of non‐malignant lesions in this region [6, 16]. Our finding, however, contrasts with a study by Vidyavathi et al., which reported higher prevalence rates of gastric and esophageal cancers [13]. The disparity in the reported prevalence of benign versus malignant upper GI lesions is largely attributable to differences in the clinical characteristics of study participants. While our study included all patients who underwent upper GI endoscopy with biopsy regardless of their endoscopic findings, Vidyavathi et al. enrolled only patients with visible mucosal lesions such as ulcers, polypoid or ulcerative growths in the upper GI tract in their study [13]. Also, the younger demographic profile of our participants, with nearly half under 50 years of age, may have contributed to the higher prevalence of benign lesions recorded in this study. Our research, just like a Pakistani study [17], identified the stomach as the most common site for endoscopic biopsy. This may be explained by the fact that the commonest indication for endoscopic biopsy among our participants was epigastric pain, and the stomach is the most common organ site for pathologies that cause epigastric pain and dyspepsia.

Our study demonstrated a high level of concordance between endoscopic and histopathological diagnoses of esophageal lesions, with esophageal biopsies showing 100% agreement for malignant conditions. The only suspected case of esophageal cancer among the participants was histologically confirmed to be squamous cell carcinoma. In some previous studies [18, 19], squamous cell carcinoma emerged as the most prevalent esophageal malignancy within the study population. Esophageal cancer is recognized as the seventh most common cancer worldwide and stands as the sixth primary cause of death related to cancer, with its burden high in less developed regions where almost 80% of cases occur [20, 21]. Esophageal squamous cell carcinoma (ESCC) represents a significant cause of mortality globally, with a particularly high incidence in Southern and Eastern Africa, as well as central Asia [22].

**Figure 2:**
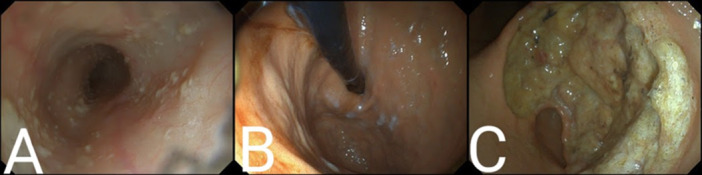
Endoscopic images showing normal esophagus (A) and cardia (B). There is a large, irregular, deep, ulcerated lesion with rolled edges in the antrum (C).

In this study, inflammatory lesions emerged as the predominant finding in gastric biopsies, with chronic gastritis being the most frequently diagnosed condition. This trend aligns with previous reports by Memon et al. and Gumber et al., who documented similar patterns [17, 19]. Gastritis with intestinal metaplasia constitutes a significant histopathological finding, indicative of a premalignant lesion. In 10% of cases, intestinal metaplasia was seen along with chronic gastritis. A comparable finding was noted by Krishnappa et al [23]. The predominance of chronic gastritis as noted in our study may be due to a number of reasons. First and foremost, the study was conducted in a Ghanaian district that is predominantly rural, with farming being the primary livelihood for the majority of the population. Farming activities require long hours of physical labor, which often causes chronic musculoskeletal pain among farmers, leading to the frequent intake of over‐the‐counter non‐steroidal anti‐inflammatory drugs (NSAIDs). Again, the demands of farming can result in irregular eating patterns among farmers. Additionally, rural dwellers are highly exposed to *Helicobacter pylori* due to poor sanitary conditions. Histological examination revealed malignancy in only 1.7% of gastric biopsies, predominantly adenocarcinoma and squamous cell carcinoma. This finding is consistent with previous studies that reported lower rates of malignancy in gastric biopsies [17, 24]. Interestingly, squamous cell carcinoma of the stomach is an exceptionally rare entity with fewer than 100 cases reported in the literature to date [25]. This unusual finding warrants further investigation, as it may represent either metastatic disease or an atypical primary tumor. Parks first proposed the diagnostic criteria for primary gastric squamous cell carcinoma, which include (i) the tumor should not extend into the esophagus, (ii) the tumor should not be located at the cardia, and (iii) there should be no evidence of squamous cell carcinoma in any other part of the body [26]. In 2011, the Japanese Gastric Cancer Association also came up with histological criteria for diagnosing primary gastric squamous cell carcinoma, which comprises the following: (i) all tumor cells must be squamous cell carcinoma cells without any gland cancer cells, and (ii) squamous cell carcinoma must originate in the gastric mucosa [27]. In the index case, endoscopy revealed a large, irregular, deep, ulcerated lesion with rolled edges located in the antrum. The lesion was covered with a thick, dirty‐yellowish exudate. The esophagus and cardia of the stomach were normal (Figure 2). Following the histological confirmation of a squamous cell carcinoma, the patient was referred to see a general surgeon and an oncologist at a tertiary hospital for further evaluation and management.

**Figure 3:**
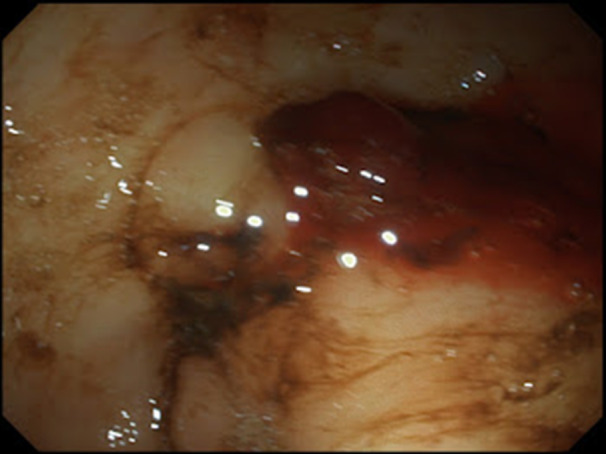
A polypoid antral lesion obscured by an adherent, dark red blood clot and recent oozing.

One of the three cases endoscopically suspected to be gastric malignancies turned out to be chronic gastritis with intestinal metaplasia after histological examination. This was detected in a 58‐year‐old male presenting with a 2‐month history of weight loss, which was associated with abdominal pain and an episode of melena. Endoscopic assessment showed a polypoid lesion with a relatively pale, smooth mucosal surface located in the antrum. A part of the lesion was obscured by an adherent, dark red blood clot and recent oozing. The luminal walls were covered with coffee‐ground residue (Figure 3).

The endoscopic findings in the duodenal area were predominantly benign. Benign duodenal ulcer was the most frequently encountered lesion in the duodenum, consistent with findings from a previous study reporting a high prevalence of duodenal ulcers [28]. Chronic duodenitis also emerged as the predominant inflammatory lesion affecting the duodenum. Similar findings have been documented in previous studies, reinforcing the consistency of this observation [24, 29, 30]. However, the lower concordance observed for duodenal ulcers (42.9%) suggests that visual inspection alone is insufficient, and therefore, a biopsy should be recommended for all duodenal lesions irrespective of their endoscopic appearance to ensure diagnostic accuracy. This recommendation is particularly important given the regenerative but vulnerable nature of the duodenal epithelium, which remains susceptible to inflammatory insults and may present with overlapping endoscopic features [17].

The sensitivity for detecting malignancy was exceptionally high at 100% (95% CI: 29.2–100), indicating that all malignant cases were correctly identified by endoscopy, though the wide confidence interval reflects the small number of malignant cases (*n* = 3). Similarly, the specificity for malignancy was 99.2% (95% CI: 95.8–100), reflecting a very low rate of false positives. For benign lesions, sensitivity was 99.2% (95% CI: 95.8–100) and specificity was 100% (95% CI: 29.2–100). The narrower interval around the high sensitivity for benign lesions underscores the reliability of upper GI endoscopy in identifying non‐malignant conditions, whereas the wider interval around the high sensitivity for malignancy emphasizes the need for cautious interpretation due to the small number of malignancies identified in this study. These findings highlight the indispensable role of histopathological confirmation, particularly in suspected malignancies where endoscopic impressions may occasionally overestimate disease presence. The PPV for malignancy was 75%, suggesting that three‐quarters of lesions identified as malignant by endoscopy were confirmed histologically. The NPV reached 100%, demonstrating that all cases deemed non‐malignant by endoscopy were indeed histologically benign. The overall diagnostic accuracy for malignancy was 99.2%. For benign lesions, the sensitivity and NPV were 99.2% and 100%, respectively, while the specificity and PPV were 100% and 75%, respectively. The diagnostic accuracy for benign lesions was also 99.2%. Not only do these findings support the reliability of upper GI endoscopy in ruling out malignancy and identifying non‐malignant lesions, but they also indicate that a proportion of endoscopically suspected malignancies may not be confirmed histologically. This underscores the importance of obtaining histopathological confirmation for definitive diagnosis, especially in cases where endoscopy suggests malignancy.

In the current study, a statistically significant association between endoscopic and histopathological diagnoses of gastric lesions was noted (*p*‐value = 0.001). Adequate patient preparation, thorough pre‐procedural assessment, employing the use of high‐definition endoscopes, meticulous mucosal inspection, targeted tissue sampling, and taking an adequate number of biopsy specimens may have accounted for this observation. A similar trend was reported in an earlier study, which found a statistically significant association between endoscopic and histopathological findings [16].

Although the overall Cohen's Kappa coefficient of 0.796 indicates a substantial level of agreement between endoscopic and histopathological diagnoses of gastric lesions, this statistic can be misleading when prevalence is extreme, as in our cohort, where 97.7% of lesions were benign. To address this, we calculated the PABAK, which was 0.966, suggesting near‐perfect agreement. The higher PABAK is an indication that the observed concordance was not merely a statistical artifact of lesion distribution but a genuine diagnostic alignment. This strengthens the reliability of upper GI endoscopy, particularly in diagnosing benign gastric conditions in resource‐limited settings where histopathology is not readily available. Our concordance exceeded that reported in several comparable studies, such as Rauta et al. [6], who found a Kappa coefficient of 0.49 despite high agreement for gastritis and esophagitis. Factors likely contributing to our higher agreement include the use of high‐definition equipment (Sonoscape HD 500 with chromoendoscopy capability), experienced endoscopists, and the predominance of benign lesions that are more readily recognized endoscopically.

The findings of this study have some epidemiological and clinical implications. The high prevalence of gastritis noted in the present study mirrors regional trends observed in Ghana and across sub‐Saharan Africa, where *Helicobacter pylori* infection and the abuse of NSAIDs remain common etiological factors for gastric inflammation. Tailored public health education programs are urgently needed to reduce the abuse of NSAIDs. In addition, clinicians should strictly adhere to guideline‐directed recommendations for *Helicobacter pylori* eradication in order to prevent long‐term gastric complications. It is also worth noting that the high level of concordance between endoscopic and histopathological findings with regard to benign GI tract diseases leads to consistent and more accurate data on the prevalence of those disorders, as the two diagnostic modalities produce similar results. Clinically, the high correlation between the two major diagnostic methods with respect to gastritis helps to streamline clinical decision‐making. This allows for prompt initiation of appropriate therapy and reduces delays associated with histological confirmation, particularly in low‐resource settings where histology services are not readily available. Unnecessary endoscopic biopsy drives up out‐of‐pocket costs and places a significant financial burden on low‐income patients in most districts across Ghana. Given the high diagnostic accuracy of endoscopy for benign conditions like gastritis, adopting a “selective‐biopsy” approach when endoscopic findings are overwhelmingly benign can translate into significant cost savings for patients and promote healthcare equity. Furthermore, the study highlights the need for histopathological confirmation to enhance diagnostic accuracy, especially for conditions with relatively lower concordance rates, such as duodenal ulcer and duodenitis.

Despite its valuable insights, this study has several limitations. Firstly, its retrospective design inherently limits control over data completeness and quality. Forty‐six patients were excluded due to unavailable histopathology reports (Figure 1). Although the missing data occurred at random and were not linked to specific lesion types, their absence may slightly limit the completeness of our findings and the precision of concordance estimates.

Secondly, the study was conducted at a single district hospital, which may limit the applicability of the findings to other regions with different demographic profiles, healthcare infrastructure or disease prevalence. The relatively small sample size of participants with available histopathology reports may also constrain statistical power, particularly in subgroup analyses. Although multiple pathologists were involved in the examination of biopsy specimens, interobserver reliability was not formally assessed, thus making it difficult to determine the degree of consistency or agreement between the pathologists. In addition, this study was constrained by the absence of an in‐house histopathology laboratory at Methodist Hospital, Wenchi. Biopsy specimens had to be processed externally, leading to increased risk of sample degradation, added transport costs, and potential delays in diagnosis as well as oncology referrals. These barriers to timely histopathological confirmation in district hospitals highlight the need for local laboratory capacity. Again, the high sensitivity of endoscopy for malignancies observed in this study cannot be generalized due to the small number of malignant cases, which heavily restricts statistical power. Lastly, the 13‐month retrospective period may be insufficient to capture potential seasonal variations in GI disease presentation or shifts in clinical practice patterns over the period.

In conclusion, our study demonstrated high diagnostic concordance between upper GI endoscopy and histopathology, particularly for benign lesions such as gastritis. The sensitivity of endoscopy for detecting malignancies was equally high, albeit for a small number of malignant cases. Duodenal lesions exhibited lower concordance rates between the two diagnostic modalities. These findings suggest that clinicians in low‐resource settings without histopathology services can safely initiate targeted treatments for benign upper GI diseases based primarily on endoscopic findings. The study also emphasizes the need for routine biopsy for all duodenal lesions regardless of their endoscopic appearance, to ensure diagnostic accuracy. Again, the findings highlight the indispensable role of histopathological confirmation in suspected cases of malignancy.

## Author Contributions

**Prosper Adjei:** conceptualization, data curation, writing – original draft, writing – review and editing. **Samuel Kyeremeh Adjei:** formal analysis, data curation, writing – original draft, writing – review and editing.

## Funding

The authors have nothing to report.

## Disclosure

All authors have read and approved the final version of the manuscript. **Samuel Kyeremeh Adjei** has full access to all of the data in this study and takes complete responsibility for the integrity of the data and the accuracy of the data analysis.

## Ethics Statement

Ethical approval was obtained from the Research Ethics Committee of Methodist Hospital, Wenchi, before commencement of the study (Ethics approval reference: MHW/RP/103/01). The study did not incorporate any identifying details such as patient name or identification number, ensuring that all data was handled with the highest level of confidentiality. Additionally, the requirement for informed consent was waived by the Research Ethics Committee.

## Conflicts of Interest

The authors declare no conflicts of interest.

## Transparency Statement

Samuel Kyeremeh Adjei affirms that this manuscript is an honest, accurate and transparent account of the study being reported; that no important aspects of the study have been omitted; and that any discrepancies from the study as planned (and, if relevant, registered) have been explained.

## Data Availability

The data that support the findings of this study are available from the corresponding author upon reasonable request.
